# Psychometric evaluation of the Arabic version of the Irish Assertiveness Scale among Saudi undergraduate nursing students and interns

**DOI:** 10.1371/journal.pone.0255159

**Published:** 2021-08-12

**Authors:** Mansour Mansour, Abd Alhadi Hasan, Ahmad Alafafsheh

**Affiliations:** 1 Fundamentals of Nursing Department, College of Nursing, Imam Abdulrahman Bin Faisal University, Dammam, Saudi Arabia; 2 Nursing Department, Fakeeh College for Medical Sciences Jeddah, Saudi Arabia; 3 Nursing Department, Al-Ghad International College for Applied Medical Sciences, Riyadh, Saudi Arabia; Universidad Nacional Autonoma de Mexico UNAM, MEXICO

## Abstract

**Background:**

Irish Assertiveness Scale is commonly used to examine the individual’ level of assertiveness. There is no adequately validated Arabic instrument that examines the level of assertiveness among Arabic-speaking undergraduate nursing students.

**Objectives:**

The purpose of this study was to translate, then evaluate the psychometric properties of the Arabic version of the Irish Assertiveness Scale among Saudi undergraduate nursing students and interns.

**Design:**

Cross-sectional survey.

**Settings:**

Three nursing colleges from three provinces in Saudi Arabia: Riyadh, Eastern and Makkah provinces.

**Participants:**

283 questionnaires were completed by 3^rd^ and 4^th^ year undergraduate nursing students, and nursing interns.

**Methods:**

A standard procedure including forward-backward translation, cultural adaptation and pilot testing was adopted to translate the Irish Assertiveness Scale into Arabic language. Content validity was measured using content validity index. Scale reliability was measured using cronbach’s alpha coefficient and mean inter-item correlation. The sample was randomly split, and exploratory and confirmatory factor analysis was then conducted on each sample to examine the construct validity of the proposed scale. A subsequent convergent validity and discriminant validity were also tested.

**Results:**

The item-level content validity index ranged from 0.9 to 1.0, and the overall content validity index was 0.93. The exploratory factor analysis resulted in 23-items, four-factor solution explaining 49.4% of the total variance. The mean inter-item correlation for each factor ranged between 0.22 and 0.4. Cronbach’s alpha coefficients for the overall scale was 0.80. The confirmatory factor analysis showed that the proposed four-factor solution had the best model fit. Whilst discriminant validity was supported in the new model, convergent validity was partially met.

**Conclusions:**

This study contributed toward establishing the Arabic version of the Irish Assertiveness Scale. Considering the limitations of the convergent validity demonstrated in the new instrument, a modified version of the Irish Assertiveness Scale might be needed to ascertain the most feasible model which best captures the level of assertiveness in Arabic cultural context.

## 1. Background

Assertive communication has become a key skill for successful interpersonal relationship, and its contributions to communication competence has increasingly been recognised [1]. Assertiveness as a social skill is defined as “… the degree to which people speak out and stand up for their own interests when they are not perfectly aligned with others” [2]. Evidence emphasizes that being assertive and able to speak up against unsafe practice is considered one of the pillars for establishing patient safety culture [3]. Patient safety is upheld when health care professionals, including nurses, are not only concerned with protecting their personal rights, but also extending that to advocating patient right, safety and welfare [4].

Student nurses are often unable to exert their assertive communication skills, particularly when challenging unsafe practice. During the ‘encounter’ phase of socialization, nursing students were said to quickly portray themselves as passive and obedient learners [5]. Challenging others’ practice is perceived as problematic for student nurses, mainly due to the imbalance of power, where instructors or academic mentors are seen as instrumental to passing or failing the student nurses in their placements [6]. But also, student nurses often perceive practicing assertively as a source of tension with other members of staff, which may lead to social isolation and even rejection from the team [5]. Therefore, student nurses tend to keep low-profile in their social encounter so as to better fit into the nursing team, particularly during their clinical placement. Utilizing empirical method to Identify the level of assertiveness among student nurses is the first step in planning any future interventions that would enable student nurses to develop fine-tuned, complex communication skills and help them to manage their feelings and communicate more assertively in their future professional roles. Several studies have examined the level of assertiveness among undergraduate nursing students. One of the widely used instruments to measure the level of individual assertiveness is the Irish Assertiveness Scale [7]. This scale has been used previously to assess the level of assertiveness among undergraduate nursing students and trainees [8–10], and it was shown to have sound psychometric properties when used among English-speaking undergraduate nursing students.

In Saudi Arabia and the Middle East, the vast majority of nursing students are native Arabic speakers. However, most of the research which examined the level of assertiveness among health care professionals has utilized English-written assertiveness surveys [11, 12], with little published evidence on a systematic transcultural adaptation and validation of the Arabic version of these surveys. Hammoud and colleaques [13] used Arabic version of the Irish Assertiveness Scale to examine the level of assertiveness among undergraduate nursing students in one university in Syria. Whilst the researchers have stated that they had translated the Irish Assertiveness Scale into Arabic language [7], there was neither an adequate description of the translation process, nor a sound psychometric assessment of the translated scale, which casts serious doubts on the credibility of the new instrument and whether it has truly examined the level of students’ assertiveness. The use of psychometrically tested assertiveness survey would provide future researchers with Arabic-written survey which has robust reliability and validity report. Therefore, the aim of this research was to examine the psychometric properties of the Arabic version of the Irish Assertiveness Scale among a sample of Saudi undergraduate nursing students and interns.

## 2. Methods

### 2.1. Design

Cross-sectional design was utilized in this research.

### 2.2. Participants, settings and sampling

All 3^rd^, 4^th^ year undergraduate nursing students and nursing interns (*n* = 570) from three Saudi Colleges of Nursing in the Eastern, Riyadh and Makkah provinces were invited to participate in this study (one public and two private institutions). To recruit the participants, a convenient sampling technique was adopted. All undergraduate nursing students and interns who met the eligibility criteria in the three locations were invited to complete an online questionnaire. To be included in the study’ sample, the participants had to be registered in a regular BSc undergraduate nursing program which are accredited by the Saudi’ Education and Training Evaluation Commission (ETC), and must be either 3^rd^ or 4^th^ year undergraduate nursing students or nursing interns. Student nurses in the three institutions must complete four years of training, before joining an accredited nursing internship program in the fifth year. Upon successful completion of the internship program, the student nurses graduate with BSc degree in nursing. They still have to pass the Saudi Commission for Health Specialties (SCHS)’ licensure examination before becoming fully practising registered nurses. For the required sample size, there are different rules of thumb in relation to the respondent-to-item ratio in validation studies, ranging from 5:1 [14] to 30:1 [15. 16]. Similarly, there are several rules-of-thumb for the required sample size for the Exploratory Factor Analysis (EFA), ranging from 3–20 participants per item [17]. Our target sample size was 140 for the EFA as absolute minimum (for 28 items scale), given the fact that the collected sample will have to be randomly split into two samples to allow for cross-validation. Therefore, we aimed to collect 280 participants.

### 2.3. Instrument

The 28-items, Irish Assertiveness Scale was used in this study [7]. Each item has 5 points-Likert scale answers, with scoring ranges from 1 (always) to 5 (never). The scale incorporates six dimensions of assertiveness: Positive assertion (expression of admiration and compliment), negative assertion (direct expressions of justified anger or disagreement), self-denial (exaggerated concern for the feelings of others), the ability to deal with criticism (constructive vs destructive management of criticism), confronting others (standing up for personal views) and the spontaneous expression of feelings (when both responding to others’ feelings or expressing own feeling) [7, 18, 19]. Published evidence suggests a satisfactory to good face validity for the instrument when tested among undergraduate nursing and midwifery students from Ireland [7], Greece [8] and Australia [9]. The instrument has also demonstrated satisfactory reliability measures using cronbach alpha (α = 0.65) [8] and moderate pearson’s correlation coefficient when assessing test–retest reliability of (*r* = 0.57).

#### 2.3.1 Transcultural adaptation: Validity and reliability

**Fig 1:**
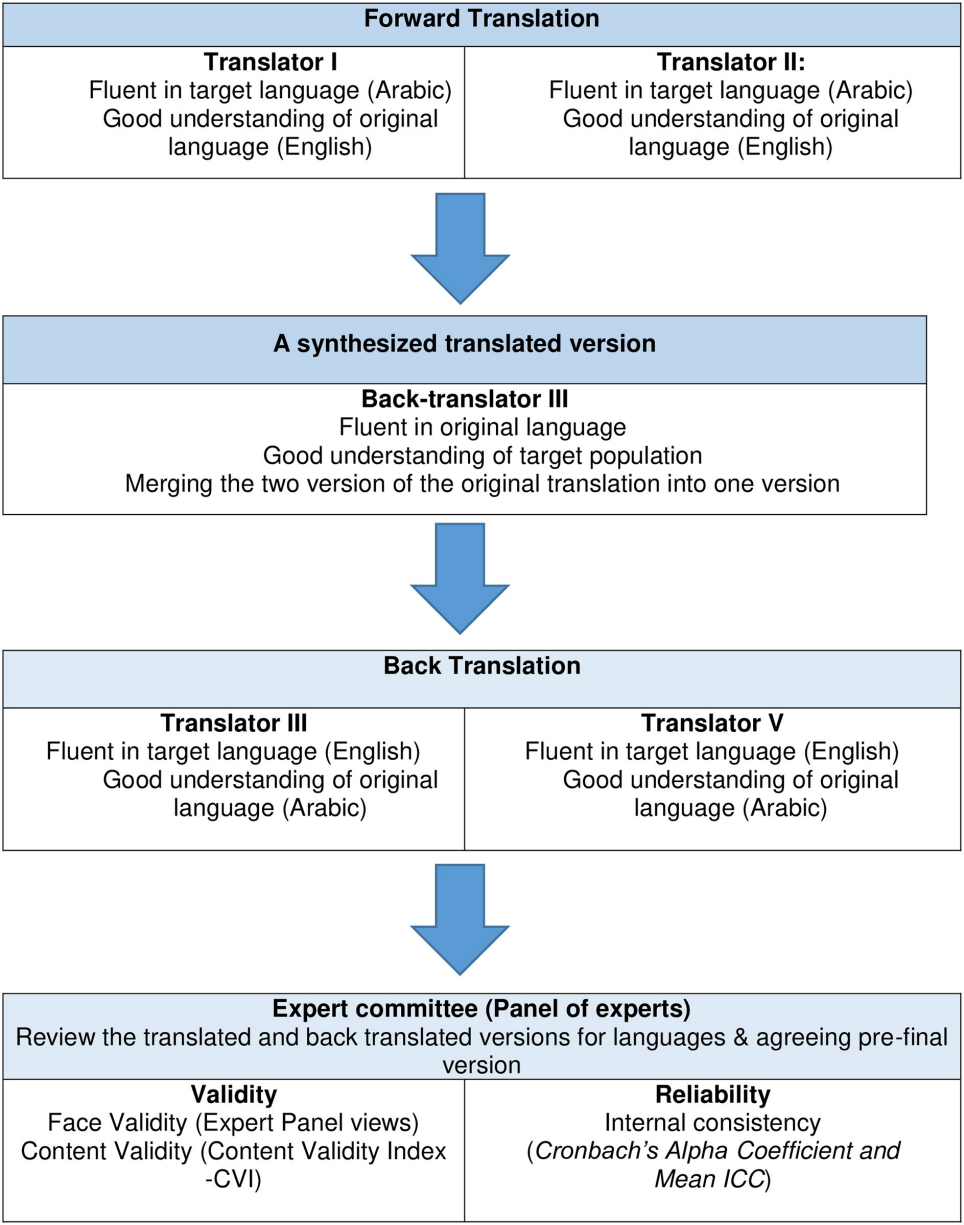
Translation framework (Beaton et al. 2000).

Permission was secured from the original authors to use the Irish Assertiveness Scale. Translating a questionnaire into a different language must accommodate the cross-cultural adaptation of the scale to maintain the equivalency between source and target language, but also to retain the psychometric properties of the original scale [20]. In the current study, the research instrument was translated based on Beaton, Bombardier [20]’ standard guidelines for translating surveys which includes: Forward and backward translation, cultural adaptation and pilot testing. The process for translating the research scale is described in Fig 1.

Firstly, the scale was translated into Arabic language by two independent translators. One was a health care professional who is heavily involved in a previous research on assertiveness among nursing workforce, and the other was a professor in linguistics studies with subspecialty in English language, who was neither aware, nor informed about the concept of the research [20]. Both translators were fluent in the Arabic language and have an excellent knowledge of the English language. The translated versions were then independently synthesized into one version (version A) by a third translator. After that, the synthesized version was back translated into Arabic language by two independent translators who were blind to the original version, and who were fluent (and native) in English and have an excellent knowledge of the Arabic language. The new translated version (version B) along with the (version A) were then submitted to an 8-members expert panel (1 methodologist, 1 health professional with PhD degree, 1 language professional with PhD degree, and the 5 translators who were directly involved in the translation process). Rubio and colleaques [21] suggested that the number of expert committee’ members should be more than 5, but less than 10. The expert panel reviewed both versions (A + B) of the scale and agreed a pre-final version for pilot testing.

The expert panel reviewed the face validity of the new scale. Each member of the panel rated each item of the pre-final version of the scale from 1 (Not relevant to the topic being researched) to 5 (Very relevant to the topic being researched). The scale was finally piloted on a group of 30 participants across the three campuses to test the readability and understanding of the questions. There was one minor change to one of the questions where additional phrases were added: when *I’m in groups*, *I take the decision*) was changed to (*when I’m in groups*, *I tend to take the decision*).

### 2.4. Data collection

An email invitation was sent by the administrative staff to all eligible students and nursing interns in each of the participating colleges. The email contained an electronic link which directs the students to the URL for the study survey. A follow up reminder email was sent to all the potential participants after one week. Sue and Ritter [22] suggested that if the participants do not respond after the first reminder using online survey, other follow up email reminders may only marginally increase the response rate or not increase it at all. Data collection was carried out between March 2019 and May 2019.

### 2.5. Ethical considerations

The first page of the survey contained the Participants Information Sheet (PIS), which emphasized the voluntary participation. The PIS stated that by completing the online survey, the participants agree to participate in this study. In each of the participating colleges, members of the research team were academics who may have been involved in delivering some form of teaching and assessment to the participating students. Therefore, it was anticipated that participants may feel vulnerable and coerced to participate in this study. For this reason, the PIS stressed that participating in this research is voluntarily, and the students do not need to provide any personal information which may make them identifiable. This was likely to make the students feel more secured and autonomous in their decision to whether to complete the survey or not. Moreover, allowing sufficient time for the students to respond is said to help avoid face-to-face contact with the research team which is likely to minimize any potential feeling of coercion to participate [23]. Prior to commencing data collection, the Institute Review Board (IRB)’ approvals were obtained from each research site. There were three IRB permissions secured before data collection commenced in each site:

1. Imam Abdulrahman Bin Faisal University’ Institute Review Board (IRB) Committee. (IRB No: IRB -2018‐ 04–319)
2. Al-Ghad International Colleges for Applied Medical Sciences’ Institutional Review Board Committee—Riyadh
3. Fakeeh College for Medical Sciences’ Institutional Review Board Committee—Jeddah. IRB No: 24/IRB/201

### 2.6. Data analysis

The participant responses were coded and analyzed using Statistical Package for Social Science (SPSS) version 24, and 11 items were reversed-coded (item no. 4,5,7,9,10,16,17,18,19,20,24). The demographic data was analyzed using descriptive statistics including frequencies, mean and standard deviations. Content validity index (CVI) was calculated for each item and for the overall scale by examining the panel members’ numerical responses to each item and dividing them on the number of raters [21]. The reliability of the scale was measured using cronbach alpha coefficient for each item and for the overall scale, with 0.7 value considered as good, and corrected-item total correlation for each item with 0.3 as the cutting off value [17]. The mean inter-item correlation (IIC) was also used to measure the reliability of subscales with less than 10 items [24]. In line with the best practice in psychometric testing and cross-validation of instruments [25], the sample was randomly split using the random function in SPSS software, and exploratory and confirmatory analysis was then conducted on each sample to examine the construct validity. Exploratory Factor Analysis (EFA) was conducted on the first split sample (*n* = 157) to explore the factor structure of the new proposed scale. The outcome items were then rearranged to build a revised factor solution. Confirmatory Factor Analysis (CFA) (*n* = 126) using AMOS 21.0 (Chicago, IL, USA) was then carried out on the second split sample (*n* = 126) to examine how closely the construct of the original scale fits into the revised factor solution using model fit indices and standardized factor loadings [26]. Several indices were used to decide the model fitness to the data where the chi-square statistic divided by the degrees of freedom (χ3/df) is ≤ 3, Tucker-Lewis index (TLI) >0.90, Comparative Fit Index (CFI) >0.90, Incremental Fit Index (IFI) > 0.90, Root Mean Square Error of Approximation (RMSEA) < 0.06 [27] and Standardized Root Mean Square Residual (SRMR) < 0.80 [16, 27, 28]. Individual factor loading of 0.3 during Confirmatory Factor Analysis was adopted as minimal level of practical significance [16]. Convergent and discriminant validity, and composite reliability of the new model were examined using Fornell and Larcker [29]’ criteria. Convergent validity was met when the average variance extracted (AVE) was at least of 0.5 for each factor. Discriminant validity was determined when the AVE is greater than maximum shared squared variance (MSV). Composite reliability (CR) was also established with 0.6 as the minimum value.

## 3. Results

### 3.1. Demographical data

283 questionnaires were completed (49.6%). Table 1 shows the demographics information of the participants.

**Table 1:** Demographics characteristics of the participants.

Demographics variables		*n*	%
Age	< 20 years old	3	(1.1)
	20–24 years old	259	(91.5)
	25–29 years old	18	(6.4)
	30 years old or older	3	(1.1)
Gender	Male	89	(31.4)
	Female	194	(68.6)
Year of study	3^rd^ year	112	(39.6)
	4^th^ year	80	(28.3)
	Nursing intern	91	(32.2)
Geographical area of institution	Eastern Province	151	(53.4)
	Riyadh	38	(13.4)
	Makkah Province	94	(33.2)

Most of the participants were Female (about 70%, *n* = 194), with age ranged between 20–24 years old (92%, *n* = 259). Almost half of the participants were recruited from one academic institution in the Eastern Province of Saudi Arabia (54%, *n* = 151). Also, 40% (*n* = 112) of the participant were 3^rd^ year students, and the remaining ones were 4^th^ year (28%, *n* = 80) and nursing interns (*n* = 92, 32.2%).

### 3.2. Content validity

The CVI for the 28 items on the Arabic version of the Irish Assertiveness Scale ranged between 0.9 and 1.0 (two items: 23 & 27 had CVI of 0.9, and the remaining 26 items had CVI of 1.0). All raters agreed on each item’ relevance to the Arabic cultural context. The CVI for the total scale was 0.93 which is considered significantly high, given that the 8 raters had to collectively agree on the relevance of each item [30].

### 3.3. Exploratory factor analysis

Exploratory Factor Analysis (EFA) using Principal Component Analysis (PCA) with Varimax Rotation and eigenvalue value of 1 [17] was conducted. The Kaiser-Meyer-Olkin Measure of Sampling Adequacy (KMO) was 0.80, and the Bartlett’s Test of Sphericity was significant (*p* = 0.000), confirming factorable sample size. The EFA was initially run freely without any constraints to factor numbers, resulting in 8 factors explaining 61.2% of the total variance. Items were retained if they had a factor loading of 0.4 or more. Subsequent Rotated Component Matrix and factor extractions suggested that four-factor structure was best. Items no.1,5,7,16,19 had zero or very poor loading on any of the factors, so there were deleted. A second EFA constrained to four factors was conducted, which accounted for 49.4% of the total variance. These factors were: *Negative Self Assertion*, *Positive Assertion*, *Confronting Others and Positive Expression*. (Table 2).

**Table 2:** Exploratory Factor Analysis (EFA) of the 23-items factor solution of the Arabic version of the Irish Assertiveness Scale.

Items	*Negative Self Assertion*	*Positive Assertion*	*Confronting others*	Positive expression	*Corrected Item-Total Correlation*	*Mean Inter-item correlation (Mean IIC)*	The Eigen value	% of variance explained
Factor Solution								
Q25 I tend to be over-apologetic to Colleagues	.78				.69	0.38	6.4	26.1
Q22 I feel uncomfortable asking friends to do favours for me	.77				.68			
Q21 At work I avoid asking questions for fear of sounding stupid	.72				.67			
Q27 I would feel uncomfortable expressing annoyance at a senior colleague	.70				.61			
Q23 When someone pays me a compliment, I feel unsure of what to say	.67				.60			
Q28 I am a follower, rather than a leader	.65				.53			
Q8 If a friend makes an unreasonable request, I would find it difficult to refuse	.64				.58			
Q12 At work I feel unsure what to say when I am praised	.65				.61			
Q6 I find criticism from friends and acquaintances hard to take	.59				.61			
Q2 I feel uncomfortable asking a colleague to do a favour for me	.60				.52			
Q9 I would feel uncomfortable paying a compliment to a junior colleague	.60				.58			
Q3 I find it difficult to compliment and praise friends and acquaintances	.58				.60			
Q13 I tend to be over-apologetic to friends and acquaintances	.53				.43			
Q26 I tend to be over-concerned about patients’ welfare	.52				.45			
Q24 If I was impressed by the actions of a senior colleague, I would tell him/her		.70			.47	0.27	1.99	8.37
Q20 If I disagreed with a decision made by a senior colleague, I would tell him/her		.60			.30			
Q17 I would ask for constructive criticism about my work		.59			.24			
Q18 When I am with friends, I am frank and honest about my feelings		.59			.34			
Q4 If a senior colleague made an unreasonable request, I would refuse			.80		.26	0.22	1.68	7.56
Q11 When I know a friend’s opinion is wrong, I would disagree with him/her			.52		.30			
Q10 If I was busy, I would ignore the demands of a senior colleague			.49		.30			
Q14 I try to avoid conflict at work				.81	0.4	0.4	1.33	7.35
Q15 I am very careful to avoid hurting other people’s feelings				.76	0.4			

*Overall scale Alpha = 0.80

** Factor loading lower than 0.4 will not be shown in the table.

### 3.4. Reliability

The remaining 23 items were retained for reliability testing. The initial reliability test for the whole scale showed an alpha coefficient of 0.80 which is a very good. Corrected-item total correlation for each factor ranged between 0.24 and 0.69, which suggests that each item correlates well with the corresponding factor. For the first factor *Negative self-assertion*, the alpha value was 0.88, which is considered very good. However, given that each of the remaining factors has only 2 to 4 items loading on each, it is inevitable that alpha value might be low anyway [17]. It was suggested that when the number of items on any subscale is fewer than 10, the mean inter-item correlation (Mean IIC) should be reported along with cronbach alpha, with optimum Mean IIC values ranging from 0.2–0.4 [24]. The Mean IIC for four factors ranged between 0.22 and 0.4, which is within the ideal range of the Mean IIC, and indicating a good internal consistency within each subscale. (Table 2).

### 3.5. Confirmatory factor analysis

**Fig 2:**
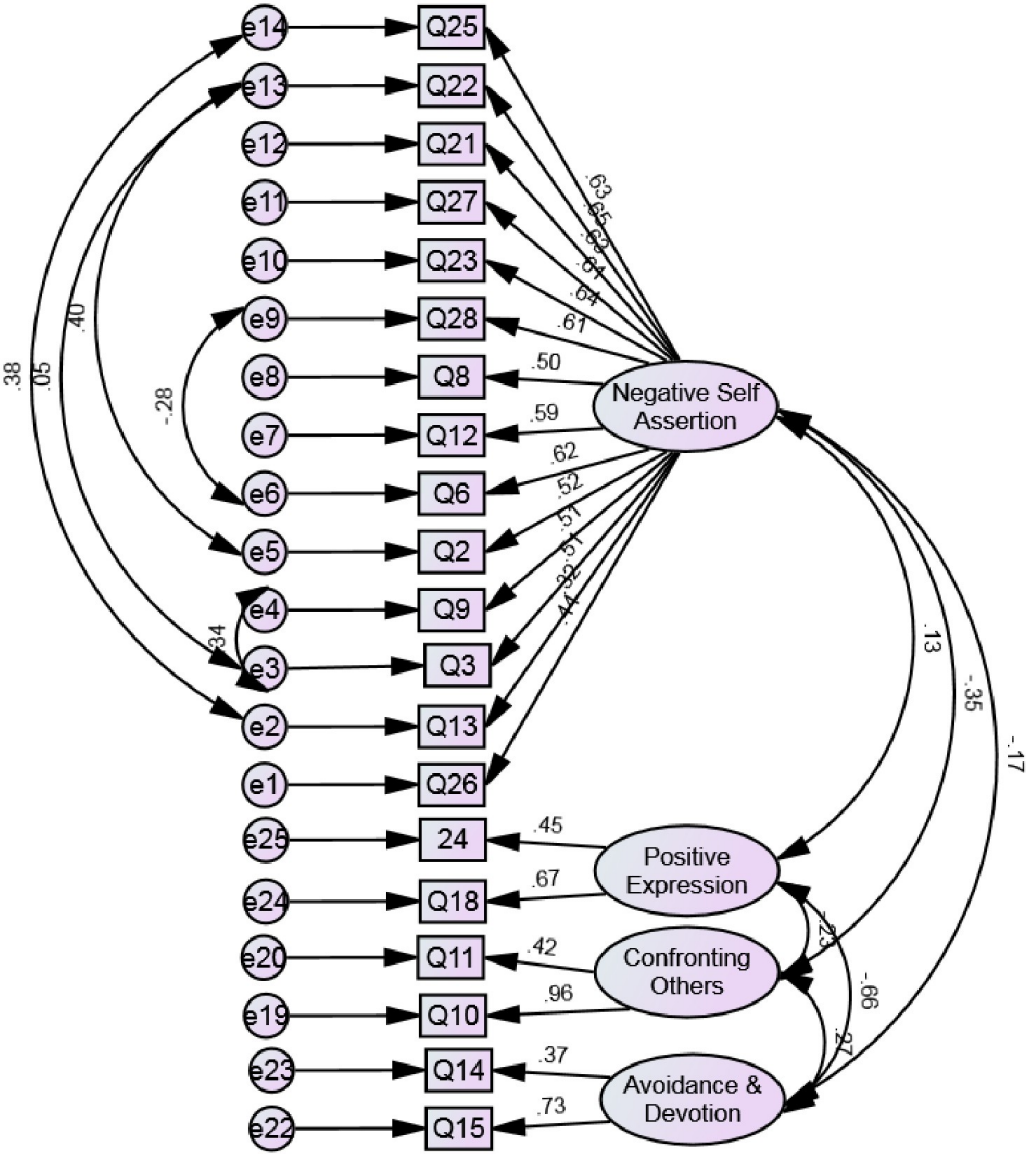
Final model of the CFA of the Arabic version of the Irish assertiveness scale.

The first Confirmatory Factor Analysis (CFA) was performed on the 23 items which resulted from the EFA factor solution. The initial model had poor fit as most of the indices were substantially below the threshold values. To improve the model fit, items no 20,17,4 were deleted as they had low item loading. Moreover, correlations among error covariance for the questions no. 22 and 3, 22 and 2, 25 and 13, 6 and 28, 3 and 9 were allowed according to the modification indices produced by the AMOS program. A second CFA was conducted on the remaining 20-item four-factor solution, which was found to have an acceptable fit, where Chi-square statistic divided by the degrees of freedom (χ3/df) = 1.44, Tucker-Lewis index (TLI) = 0.86, Comparative Fit Index (CFI) = 0.89, Incremental Fit Index (IFI) = 0.89, Root Mean Square Error of Approximation (RMSEA) = 0.06 and Standardized Root Mean Square Residual (SRMR) = 0.08 (Fig 2).

All standardized item-factor loadings exceeded the threshold of.30. The composite reliability values for the first and third factors were 0.87, and 0.70, implying a very good and good reliability respectively [29]. But for the second and fourth factors, it was close to acceptable (0.5 each). The AVE for the third factor was 0.55, which is more than the recommended AVE cut-off point of 0.5. However, the remaining three factors have inadequate AVE measures (0.32,0.33, and 0.34 respectively), so the convergent validity was partially met. For all four factors, the AVE was higher than the MSV, hence the discriminant validity was supported (Table 3).

**Table 3:** Convergent validity, discriminant validity and composite reliability of the final CFA model of Arabic version of the Irish Assertive Scale.

Latent Variables	CR	AVE	MSV
Negative Self Assertion	0.87	0.32	0.12
Positive Assertion	0.5	0.33	0.44
Confronting Others	0.7	0.55	0.12
Positive expression	0.5	0.34	0.44

**CR**: Composite Reliability. **AVE**: Average Variance Extracted. **MSV**: Maximum Shared Variance.

## 4. Discussion

This paper presents a translated and psychometrically tested instrument which assesses the level of perceived assertiveness among Arabic-speaking population. We conducted a forward and backward translation of the instrument, and the CVI for the translated scale was relatively high (93%), with all but two items scoring 1. This further ascertains the robustness and the broad consensus on the translation and backtranslation process, given the fact that there were 8 raters who had to agree on the translation of all items.

Upon conducting EFA and CFA, the four-factor structure was different from the six-factor structure presented in the original Irish Assertiveness Scale [7]. 8 items from the original scale had to be deleted: 5,7 and 16 (Positive assertion), 4,20,19 (Confronting others), 17 (ability to deal with criticism) and 1 (the spontaneous expression of feelings). Our aim when conducting EFA was to acquire a reasonable model that is easily understood and provides theory-driven and conceptually understandable solution with meaningful structure [31], before verifying this model using CFA. Such discrepancies between the original and the new factor solutions are not uncommon, and there is evidence of similar trend in the literature, particularly when the new translated scale is tested in different cultural contexts [20, 32]. Furthermore, the reader must be cognisant of the cultural differences between the student nurses in Ireland and Saudi Arabia. The cultural context in Saudi Arabia is heavily influenced by religion, besides, the phenomenon of assertiveness is uniquely contextualised in the Saudi Society and the wider Arabic culture, where individuals have more concerns toward how to fit into the community, respect for the tradition and indirect assertiveness [33]. In comparison with the Irish culture, such distinctively cultural norms may have been reflected in the student’s responses and the net factor structure.

It is noteworthy that there were 11 items which were written in the negative manner and had to be reversed coded, 7 of them were deleted (out of the 8 items which had to be eventually deleted) either because they haven’t loaded well or loaded poorly on any factor in the EFA and CFA. This may bring into light the likely impact of the negatively worded phrases on the overall scale’ factorability and reliability. Although the use of combined positively and negatively worded items is established in the literature to control response-style bias, recent empirical evidence suggests that such practice is not problem-free, and that reliability of the test is flawed and the unidimensionality of the test is jeopardised by secondary sources of variance when using combined positive and reversed-coded items [34]. Moreover, it was reported that item rewording can lead to distorting the factor structure of the scale [35]. In our study, all remaining reverse-coded items (4,10,11,17,18,20,24) were distinctively loading on two factors: *The positive Assertion* and *Confronting Others*, while the other remaining regular worded items were loading on the other two factors: *Negative Self Assertion* and *Avoidance and Devotion*. AlNajjar and Dodeen [36] examined the effect of items rewording on the factor structure of the Arabic version of the UCLA Loneliness Scale and found that the scale reflected two main factors clearly divided by positively and negatively worded items. The researchers called for reconsideration for the use of combined negative and positive items. The new factor structure in our study, however, correlates with the overall theoretical framework for the practice and research on assertive communication skills among undergraduate nursing students [7], and had an overall alpha value of 0.80 during the EFA, which suggests a good internal consistency among the overall items in the new instrument.

Findings from the CFA demonstrated that four-factor structure was the best model fit that is gleaned from the EFA. The composite reliability was relatively acceptable, but the convergent validity was partially supported in the CFA. This can be explained by the relatively low factor loading for some items. There are 5 items which loaded less than 0.5, but crossed the pre-defined 0.3 threshold (i.e. items no. 26, 13, 11, 14, 24). Item loading of less than 0.5 was reported to impact negatively on the AVE and composite reliability [37, 38]. Similarly, wide variation of items loading on the same factor can influence the AVE values, hence the convergent validity [39]. For example, items no. 14 and 15 have item loading of 0.37 and 0.73 respectively.

It is generally recommended that CFA is conducted on factor solutions with three or more items per factor [40]. The three factors: *Positive Expression*, *Confronting Others*, *Avoidance* and *Devotion*, had only two items loading on each. However, Worthington and Whittaker [41] reported that it is possible to retain a two-items factor when the conceptual interpretability supported a definitive two items factor retention criterion. The four-factor solution which resulted from the EFA in this study was retained as it was interpreted meaningfully in relation the overall theoretical and conceptual framework of examining level of assertiveness.

## 5. Limitations

The student nurses and interns recruited in this study came from only three academic institutions, clearly underrepresenting other undergraduate nursing students and interns in Saudi Arabia. As the students were recruited from different academic levels, they may have heterogenous experience of assertiveness, with subsequent implications on the validity of the findings, although the cronbach alpha was very good for the final Arabic version of the Irish Assertiveness Scale. Due to both time and logistical challenges, the research team was unable to conduct test-retest reliability estimates to further ascertain the psychometric properties of the new scale. However, the geographical diversity of the locations where the participants were recruited from is likely to provide sound comparability between the participating academic institutions. Because of the perceived power imbalance between the students and the academic staff in this study, some students might have felt coerced to participate, and although the research team have adopted few strategies to minimize such feeling, it may have not been completely neutralized, with potential impact on the quality of students’ responses.

## 6. Conclusion

This study contributed toward establishing the Arabic version of the Irish Assertiveness Scale. The new 20-items instrument showed stability in four-factor solution, acceptable model fit, acceptable composite reliability and discriminant validity but partially supported convergent validity. Considering the limitations of the convergent validity demonstrated in this instrument, a modified version of the Irish Assertiveness Scale might be needed to ascertain the most feasible model which best captures the level of assertiveness in Arabic cultural context. Future research is also needed to determine the impact of using positively worded items on the items loading and the subsequent reliability and validity of the scale.

## Supporting information

S1 Data (SAV)

S1 File (DOC)

S2 File (PDF)
