# Supplementary material for: Psychometric evaluation of the Arabic version of the Irish Assertiveness Scale among Saudi undergraduate nursing students and interns
Source: PLoS One. 2021 Aug 12;16(8):e0255159. doi: 10.1371/journal.pone.0255159 (PMC8360376; doi:10.1371/journal.pone.0255159)
Supplement: S1 File — (DOC) [file pone.0255159.s002.doc]

**Please mark the following questions according to the way that you would usually respond**

**Always Often Rarely Never**

1. At work I tend to keep my feelings

to myself

_________________________________________________________________

2. I feel uncomfortable asking a

colleague to do a favour for me

_________________________________________________________________

3. I find it difficult to compliment and

praise friends and acquaintances

_________________________________________________________________

4. If a senior colleague made an

unreasonable request, I would

refuse _________________________________________________________________

5. I enjoy starting conversations with

acquaintances and strangers

_________________________________________________________________

6. I find criticism from friends and

acquaintances hard to take

_________________________________________________________________

7. I want to know what my rights are

in the work situation

_________________________________________________________________

8. If a friend makes an unreasonable

request, I would find it difficult to

refuse

_________________________________________________________________

9. I would feel uncomfortable paying

a compliment to a junior colleague

_________________________________________________________________

10. If I was busy, I would ignore the

demands of a senior colleague

_________________________________________________________________

11. When I know a friend’s opinion is

wrong, I would disagree with

him/her

_________________________________________________________________

**Please mark the following questions according to the way that you would usually respond**

**Always Often Rarely Never**

12. At work I feel unsure what to say

when I am praised

_________________________________________________________________

13. I tend to be over-apologetic to

friends and acquaintances

_________________________________________________________________

14. I try to avoid conflict at work

_________________________________________________________________

15. I am very careful to avoid hurting

other people’s feelings

_________________________________________________________________

16. In a group I make the decisions

_________________________________________________________________

17. I would ask for constructive

criticism about my work

_________________________________________________________________

18. When I am with friends, I am

frank and honest about my feelings

_________________________________________________________________

19. If a colleague upsets a patient, I

would challenge him/her about it

_________________________________________________________________

20. If I disagreed with a decision made

by a senior colleague, I would tell

him/her

_________________________________________________________________

21. At work I avoid asking questions

for fear of sounding stupid

_________________________________________________________________

22. I feel uncomfortable asking friends

to do favours for me

_________________________________________________________________

23. When someone pays me a

compliment, I feel unsure of what

to say

_________________________________________________________________

24. If I was impressed by the actions of a

senior colleague, I would tell

him/her

_________________________________________________________________

**Please mark the following questions according to the way that you would usually respond**

**Always Often Rarely Never**

25. I tend to be over-apologetic to

colleagues

_________________________________________________________________

26. I tend to be over-concerned about

patients’ welfare

_________________________________________________________________

27. I would feel uncomfortable

expressing annoyance at a senior

colleague

_________________________________________________________________

28. I am a follower, rather than a

leader

Scoring:

Questions 4, 5, 7, 10, 11, 16,17,18,19, 20, 24, are scored so that “Always” receives 4 marks, “Often” gets 3, “Rarely” is 2, and “Never” receives 1

Questions 1, 2, 3, 6, 8, 9, 12, 13 14, 15, 21, 22, 23, 25, 26, 27, 28 are reverse-scored so that “Always” receives 1 mark, “Often” gets 2, “Rarely” is 3, and “Never” receives 4

The higher the final total score is, the higher that person’s level of assertiveness is deemed to be.

____________________TIPI

Scoring:

Questions 7,, are scored so that

1= Disagree strongly
2 = Disagree moderately
3 = Disagree a little
4 = Neither agree nor disagree
5 = Agree a little
6 = Agree moderately
7 = Agree strongly

“Always” receives 4 marks, “Often” gets 3, “Rarely” is 2, and “Never” receives 1

Questions 1, 2, 3, 6, 8, 9, 12, 13 14, 15, 21, 22, 23, 25, 26, 27, 28 are reverse-scored so that “Always” receives 1 mark, “Often” gets 2, “Rarely” is 3, and “Never” receives 4
