# Supplementary material for: Psychometric evaluation of the Arabic version of the Irish Assertiveness Scale among Saudi undergraduate nursing students and interns
Source: PLoS One. 2021 Aug 12;16(8):e0255159. doi: 10.1371/journal.pone.0255159 (PMC8360376; doi:10.1371/journal.pone.0255159)
Supplement: S2 File — (PDF) [file pone.0255159.s003.pdf]

هل يمكن للسمات الشخصيه لطلاب و طالبات التمريض الجامعيين السعوديين أن تتنبأ بمستوى الحزم لديهم في بيئة العمل؟ دراسه مسحيه عبر الإنترنت

الطلاب / الطالبات الاعزاء: أنتم مدعوون للمشاركة في هذه الدراسة البحثية بعنوان **هل يمكن للسمات الشخصية لطلاب وطالبات التمريض الجامعيين السعوديين أن تتنبأ بمستوى الحزم لديهم في بيئة العمل؟ دراسة مسحيه عبر الإنترنت**

ما هو غرض الدراسة؟

الهدف من هذه الدراسة هو فحص العلاقة بين مستوى الحزم لطلاب وطالبات التمريض الجامعيين السعوديين وخصائصهم الشخصية المعلنة. تكتسي آراؤك وتجاربك أهمية خاصة في فهم إذا كان يمكن للسمات الشخصية لطلاب وطالبات التمريض الجامعيين السعوديين أن تتنبأ بمستوى الحزم لديهم في بيئة العمل

ماذا سيحدث لنتائج الدراسة؟

سيتم إجراء الدراسة في ثلاث كليات تمريض: في الدمام (كلية التمريض - جامعة الإمام عبد الرحمن بن فيصل/الباحث الرئيس: د. منصور منصور)، الرياض (قسم التمريض - كلية الغد للعلوم الطبية التطبيقية/الباحث المشارك: د. احمد العفافشه) وجدة (قسم التمريض - كلية فقيه للعلوم الطبية/الباحث المشارك: د. عبد الهادي حسن). عند الانتهاء من الدراسة، سيتم تقديم تقرير عن نتائج هذه الدراسة لكليات واقسام التمريض الثلاث. كما سيتم تقديم النتائج للنشر في المجلات العلمية ويمكن تقديمها في المؤتمرات الوطنية والدولية.

لماذا تم اختيارك للمشاركة في الدراسة؟

تهدف الدراسة إلى الحصول على آراء طلاب وطالبات التمريض الجامعيين السعوديين من ثلاثة مستويات (السنة الثالثة، السنة الرابعة، والمتدربين في التمريض) بالنسبة لمستوى الحزم لديهم عند التعامل مع الاخرين والسمات الشخصية لهم المبلغ عنها، واستكشاف أي علاقة بينهم. كطالب تمريض في مرحلة ما قبل التخرج يستوفي معايير اختيار البحث، فأنت مؤهل للمشاركة في الدراسة

هل يجب علي المشاركة؟

مشاركتك في الدراسة طوعية. من خلال إكمال هذا الاستبيان وتقديمه عبر الإنترنت، فإنك توافق على المشاركة في هذه الدراسة

ما هي المساوئ والمخاطر المحتملة للمشاركة؟

لا توجد مخاطر جسدية من المشاركة في الدراسة. لا توجد احتياطات خاصة يتعين عليك اتخاذها قبل أو أثناء أو بعد المشاركة في الدراسة. لن تؤثر مشاركتك أو عدم مشاركتك في هذه الدراسة أبداً، في أي حال، على تقييمك الأكاديمي أو حقوقك أو مخصصاتك أو استحقاقاتك كطالب او طالبة تمريض.

هل مشاركتي سرية؟

المعلومات التي تقدمها ستبقى مجهولة الهوية. لا يُطلب منك وضع اسمك أو أي معلومات تتعقب هويتك عند تعبئة الاستبيان

من قيم هذه الدراسة؟

تمت مراجعة الدراسة من قبل لجان اخلاقيات البحث العلمي في جامعة الإمام عبد الرحمن بن فيصل، كلية الغد الدولية للعلوم الطبية التطبيقية وكلية الفقيه للعلوم الطبية

ما الذي يجب علي فعله إذا كنت أرغب في المشاركة في الدراسة؟

قم بتعبئة الاستبيان ادناه. سيستغرق حوالي 10 دقائق من وقتك

في العمل، اميل للحفاظ على مشاعري لنفسي

دائما	غالبا	نادرا	أبدا
◯	◯	◯	◯

.1

◯ Option 1

◯ Option 2

◯ Option 3

◯ Option 4

---

*أشعر بعدم الارتياح عندما أطلب من زميل او زميله لي أن يقدم لي خدمة

دائما	غالبا	نادرا	أبدا
○	○	○	○

*أجد صعوبة في المجاملة والثناء على الأصدقاء والمعارف

دائما	غالبا	نادرا	أبدا
○	○	○	○

*إذا تقدم أحد الزملاء او الزميلات الأعلى رتبة بطلب غير منطقي، فسوف أرفضه

دائما	غالبا	نادرا	أبدا
○	○	○	○

*أنا أستمتع ببدء المحادثة مع المعارف والغرباء

دائما	غالبا	نادرا	أبدا
○	○	○	○

*يصعب علي تقبل الانتقادات من الأصدقاء والمعارف

دائما	غالبا	نادرا	أبدا
○	○	○	○

*أرغب بالتعرف على حقوقي في مكان العمل

دائما	غالبا	نادرا	أبدا
○	○	○	○

* إذا طلب مني أحد الأصدقاء طلبا غير منطقي، اجد صعوبة في رفضه

دائما	غالبا	نادرا	أبدا
◯	◯	◯	◯

* اشعر بعدم الارتياح عند مجاملة زميل او زميله اقل رتبه

دائما	غالبا	نادرا	أبدا
◯	◯	◯	◯

* إذا كنت مشغولاً، سأتجاهل مطالب الزميل او الزميله الأعلى رتبة

دائما	غالبا	نادرا	أبدا
◯	◯	◯	◯

* عندما أعرف أن أحد الأصدقاء على خطأ ، فإنني سوف أختلف معه

دائما	غالبا	نادرا	أبدا
◯	◯	◯	◯

* في العمل، أشعر أنني غير متأكد مما سأقول عندما يتم الثناء علي

دائما	غالبا	نادرا	أبدا
◯	◯	◯	◯

* أميل إلى أن أكون أكثر اعتذارًا للأصدقاء والمعارف

دائما	غالبا	نادرا	أبدا
◯	◯	◯	◯

* أنا أحاول تجنب الصراع في العمل

دائما	غالبا	نادرا	أبدا
○	○	○	○

* أنا حريص جدًا على تجنب إيذاء مشاعر الآخرين

دائما	غالبا	نادرا	أبدا
○	○	○	○

* عندما أكون في مجموعه, أفضّل القيام بإتخاذ القرارات

دائما	غالبا	نادرا	أبدا
○	○	○	○

* أنا أطلب النقد البناء حول أدائي في العمل

دائما	غالبا	نادرا	أبدا
○	○	○	○

* عندما أكون مع الأصدقاء، فأنا صريح وصادق في مشاعري

دائما	غالبا	نادرا	أبدا
○	○	○	○

* إذا أزعج أحد الزملاء او الزميلات مريضًا، فسوف اعترض على ذلك

دائما	غالبا	نادرا	أبدا
○	○	○	○

* إذا لم أوافق على قرار صادر عن أحد الزملاء او الزميلات الأعلى رتبة ، فأنني سأخبره او أخبرها بذلك

دائما	غالبا	نادرا	أبدا
◯	◯	◯	◯

* في العمل أتجنب طرح الأسئلة خوفا من أن أبدو غبيا

دائما	غالبا	نادرا	أبدا
◯	◯	◯	◯

* أشعر بعدم الارتياح عندما أطلب من أصدقائي تقديم خدمة لي

دائما	غالبا	نادرا	أبدا
◯	◯	◯	◯

* عندما يجاملني أحد ما، أشعر بعدم التأكد مما سأقول

دائما	غالبا	نادرا	أبدا
◯	◯	◯	◯

* إذا أعجبت بتصرفات زميل او زميله أعلى رتبة، فإنني سأخبره او أخبرها بذلك

دائما	غالبا	نادرا	أبدا
◯	◯	◯	◯

* أميل إلى الإفراط في الاعتذار الى الزملاء او الزميلات

دائما	غالبا	نادرا	أبدا
◯	◯	◯	◯

* أنا أميل إلى أن أكون أكثر قلقا بشأن رعاية المرضى

دائما | غالبا | نادرا | أبدا

* أشعر بعدم الارتياح للإعراب عن الانزعاج من زميل او زميله اعلى رتبة

دائما | غالبا | نادرا | أبدا

* انا تابع, اكثر من كوني قائدا

دائما | غالبا | نادرا | أبدا

* أرى نفسي منفتح اجتماعيا، متحمس

أوافق بشدة | اتفق باعتدال | اتفق قليلا | لا أوافق ولا أرفض | لا أوافق بشكل معتدل | لا أوافق قليلا | غير موافق وبشدة

* أرى نفسي انتقادي، مشاكس

أوافق بشدة | اتفق باعتدال | اتفق قليلا | لا أوافق ولا أرفض | لا أوافق بشكل معتدل | لا أوافق قليلا | غير موافق وبشدة

* أرى نفسي جدير بالثقة، منضبط

أوافق بشدة	اتفق باعتدال	اتفق قليلا	لا أوافق ولا أرفض	لا أوافق بشكل معتدل	لا أوافق قليلا	غير موافق وبشدة
○	○	○	○	○	○	○

* أرى نفسي قلق، أنزعج بسهولة

أوافق بشدة	اتفق باعتدال	اتفق قليلا	لا أوافق ولا أرفض	لا أوافق بشكل معتدل	لا أوافق قليلا	غير موافق وبشدة
○	○	○	○	○	○	○

* أرى نفسي منفتح لتجارب جديدة، مغامر

أوافق بشدة	اتفق باعتدال	اتفق قليلا	لا أوافق ولا أرفض	لا أوافق بشكل معتدل	لا أوافق قليلا	غير موافق وبشدة
○	○	○	○	○	○	○

* أرى نفسي كتوم، هادئ

أوافق بشدة	اتفق باعتدال	اتفق قليلا	لا أوافق ولا أرفض	لا أوافق بشكل معتدل	لا أوافق قليلا	غير موافق وبشدة
○	○	○	○	○	○	○

* أرى نفسي عاطفي، رقيق

أوافق بشدة	اتفق باعتدال	اتفق قليلا	لا أوافق ولا أرفض	لا أوافق بشكل معتدل	لا أوافق قليلا	غير موافق وبشدة
○	○	○	○	○	○	○

* أرى نفسي غير منظم، مهمل

أوافق بشدة	اتفق باعتدال	اتفق قليلا	لا أوافق ولا أرفض	لا أوافق بشكل معتدل	لا أوافق قليلا	غير موافق وبشدة
◯	◯	◯	◯	◯	◯	◯

* أرى نفسي هادىء، مستقر عاطفيا

أوافق بشدة	اتفق باعتدال	اتفق قليلا	لا أوافق ولا أرفض	لا أوافق بشكل معتدل	لا أوافق قليلا	غير موافق وبشدة
◯	◯	◯	◯	◯	◯	◯

* أرى نفسي تقليدي، غير مبدع

أوافق بشدة	اتفق باعتدال	اتفق قليلا	لا أوافق ولا أرفض	لا أوافق بشكل معتدل	لا أوافق قليلا	غير موافق وبشدة
◯	◯	◯	◯	◯	◯	◯

* الجنس ?

◯ ذكر

◯ أنثى

* العمر

◯ أصغر من 20 عامًا

◯ 20 - 24 سنة

◯ 25 - 29 سنة

◯ 30 عامًا أو أكثر

*السنه الدراسيه الحاليه :

- السنه الثالثه
- السنه الرابعه
- سنة الامتياز

*أسم الكليه / الجامعه التي تدرس فيها:

- جامعة الإمام عبدالرحمن بن فيصل - الدمام
- كلية الغد للعلوم الطبيه التطبيقيه - الرياض
- كلية فقيه للعلوم الطبية - جدة

إذا كان لديك أي سؤال عن هذه الدراسه، الرجاء الاتصال على أحد العناوين التاليه

د. منصور منصور
. الباحث الرئيس أستاذ مشارك للتمريض قسم أساسيات التمريض. كلية التمريض. جامعة
الإمام عبدالرحمن بن فيصل تلفون: 0133331623
البريد الإلكتروني: mjmansour@iau.edu.sa:

د. احمد العفافشه
باحث مشارك. استاذ مساعد. قسم التمريض، كلية الغد للعلوم الطبيه التطبيقيه - الرياض
- المملكة العربية السعودية: تلفون: 0505397917
البريد الإلكتروني: aalafafsheh@gc.edu:

د. عبد الهادي حسن
. باحث مشارك. أستاذ مساعد في تمريض الصحة النفسية. كلية فقيه للعلوم الطبية -جدة-
المملكة العربية السعودية. تلفون 0568441175
البريد الإلكتروني: aalhasan@fakeeh.care:
